# Unravelling the heterogeneity and dynamic relationships of tumor‐infiltrating T cells by single‐cell RNA sequencing analysis

**DOI:** 10.1002/JLB.6MR0320-234R

**Published:** 2020-04-09

**Authors:** Xin Yu, Lei Zhang, Ashutosh Chaudhry, Aaron S. Rapaport, Wenjun Ouyang

**Affiliations:** ^1^ Department of Inflammation and Oncology Amgen Research, Amgen Inc. South San Francisco California USA; ^2^ Beijing Advanced Innovation Center for Genomics Peking‐Tsinghua Center for Life Sciences Peking University Beijing China

**Keywords:** cancer microenvironment, exhausted T cells, memory T cells, regulatory T cells, single‐cell RNA sequencing, TCR‐based lineage tracing, tumor‐infiltrating T cells

## Abstract

T cells are crucial for the success of immune‐based cancer therapy. Reinvigorating antitumor T cell activity by blocking checkpoint inhibitory receptors has provided clinical benefits for many cancer patients. However, the efficacy of these treatments varies in cancer patients and the mechanisms underlying these diverse responses remain elusive. The density and status of tumor‐infiltrating T cells have been shown to positively correlate with patient response to checkpoint blockades. Therefore, further understanding of the heterogeneity, clonal expansion, migration, and effector functions of tumor‐infiltrating T cells will provide fundamental insights into antitumor immune responses. To this end, recent advances in single‐cell RNA sequencing technology have enabled profound and extensive characterization of intratumoral immune cells and have improved our understanding of their dynamic relationships. Here, we summarize recent progress in single‐cell RNA sequencing technology and current strategies to uncover heterogeneous tumor‐infiltrating T cell subsets. In particular, we discuss how the coupling of deep transcriptome information with T cell receptor (TCR)‐based lineage tracing has furthered our understanding of intratumoral T cell populations. We also discuss the functional implications of various T cell subsets in tumors and highlight the identification of novel T cell markers with therapeutic or prognostic potential.

## Graphical abstract

Review on single‐cell RNA sequencing, coupled with TCR‐based lineage tracing analysis, with implications for biomarker identification and next‐generation cancer immunotherapies.

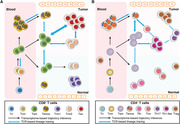

**Abbreviations**
CPI: checkpoint inhibitor
CyTOF: cytometry by time‐of‐flight
MHC: major histocompatibility complex
MSI: microsatellite‐instable
NSCLC: non‐small‐cell lung cancer
PBMC: peripheral blood mononuclear cells
PD‐1: programmed cell death protein 1
scRNA‐seq: single‐cell RNA sequencing
STARTRAC: single T cell analysis by RNA sequencing and TCR tracking
TME: tumor microenvironment
T_em_: effector memory T cell
T_exh_: exhausted T cell
T_mem_: memory T cell
Treg: regulatory T cell
T_rm_: residential memory T cell
UC: ulcerative colitis

## 1 INTRODUCTION

T cells play key roles in immune defense against tumor development and metastasis. Cytotoxic CD8^+^ T cells, CD4^+^ T helper (Th) cells (especially Th1 cells), and regulatory T cells (Treg) cells orchestrate antitumor T cell responses to fight malignancies in coordination with the rest of immune system. Harnessing antitumor T cell responses to fight malignancies has been the major focus of cancer immunotherapy. The essential antitumor effects of T cells are seen in the fraction of patients who develop long‐lasting complete responses after treatment with checkpoint inhibitors (CPIs) targeting CTLA4 and programmed cell death protein 1 (PD‐1).^1^, ^2^, ^3^ However, response rates to CPIs are not uniform, and a substantial fraction of cancer patients do not respond at all.^1^, ^2^ Better understanding of tumor‐infiltrating T cell populations and their differential responses to CPIs will be fundamental to the development of novel therapeutic strategies to enhance and broaden T cell‐mediated antitumor immune responses.

Transcriptome‐scale analysis of tumor samples is a powerful tool to reveal the molecular pathways and cellular composition of cancers. Bulk RNA sequencing of tumors can reveal whether a given tumor type or a specific cancer sample contains a high degree of T cell infiltration, and whether these T cells express higher effector or exhaustion markers.^4^ However, this type of approach is not sensitive enough to fully elucidate the factors underlying the success or failure of CPI treatments. The tumor microenvironment (TME) includes multiple heterogeneous T cell types, among many other immune and non‐immune cells, so novel multiplex and high throughput technologies are necessary to better dissect its cellular and molecular compositions. Here, we describe the recent development of single‐cell RNA sequencing (scRNA‐seq) technologies, which has become a key tool in efforts to unravel the complexities of the TME, and discuss how its applications are deepening our understanding of tumor‐infiltrating T cell populations in various human cancers.

## 2 SINGLE‐CELL SEQUENCING TECHNOLOGIES

Single‐cell resolution of cellular diversities and trajectories is critical to our understanding of immune responses to different pathogens or antigens.^5^ Over time, major technologies, including flow cytometry, in situ histological assays, and microscopy, have been developed and broadly used to categorize immune cell subsets, as well as characterize their functional phenotypes and spatial distributions.^6^, ^7^, ^8^, ^9^ Although these technologies provide invaluable insights when analyzing limited samples with a few prior selected markers, they are not suitable for dissecting heterogenous cell population in tissue and tumor in a comprehensive and unbiased manner. More recently, mass cytometry (also known as Cytometry by Time‐Of‐Flight, CyTOF) was developed to simultaneously detect more than 40 protein markers in millions of individual cells.^10^, ^11^ CyTOF has been instrumental to our understanding of TME complexity.^12^ For example, three recent studies employed CyTOF to study immune cells from patients with non‐small cell lung cancer (NSCLC), renal cancer, and breast cancer, revealing diverse lymphoid and myeloid cell populations and linking specific immune signatures with clinical features (Table 1).^13^, ^14^, ^15^ Another recent study showed that mass cytometry can be used in combination with MHC‐tetramers to analyze antigen (Ag)‐specific T cells, elucidating phenotypic differences between tumor‐ and viral‐specific CD8^+^ T cells.^16^ However, like the traditional technologies, CyTOF also has notable limitations: it requires prior knowledge to select markers, there is a lack of high‐quality reagents for certain markers, and it is relatively low‐throughput compared to genome‐wide analyses. Despite these limitations, CyTOF and emerging technologies like imaging mass cytometry^17^, ^18^, ^19^ can rapidly provide essential information on protein expression in the context of anatomical location. Data from these approaches will continue to be widely used to confirm cellular subsets, delineate cell‐cell interactions and spatial relationships, and explore clinical biomarkers.

### 2.1 The advantages and limitations of scRNA‐seq approaches

In 2009, scRNA‐seq was established to obtain unbiased appreciation of the whole‐transcriptome from a single mouse blastomere.^20^ Since then, the technology has been used to probe cellular populations as varied as differentiating embryonic cells, intracranial neurons, malignant tumor cells, and individual immune cells.^9^, ^21^, ^22^, ^23^, ^24^, ^25^, ^26^ All the while, the sensitivity, scale, and accuracy of scRNA‐seq has expanded and improved exponentially. Today, there are many single‐cell sequencing protocols available to researchers, each offering distinct advantages and disadvantages. Plate‐based approaches can profile hundreds or thousands of single cells using full‐length sequencing protocols (e.g., STRT‐seq, Smart‐seq, and Smart‐seq2),^27^, ^28^, ^29^ or pooled 3′ end sequencing approaches (e.g., CEL‐seq and MARS‐seq).^30^, ^31^, ^32^ Droplet‐based protocols (e.g., Drop‐seq, InDrop and 10× Chromium Genomics)^33^, ^34^, ^35^ and other massively parallel approaches (e.g., Seq‐well, sci‐RNA‐seq, and SPLiT‐seq) have also been developed to increase throughput.^36^, ^37^, ^38^ Comprehensive comparisons of these approaches have been reviewed elsewhere and thus will not be the focus here.^25^, ^39^, ^40^

As scRNA‐seq technologies advance, we can expect that such technologies will be widely used to uncover key processes and critical pathways in different immune cells under steady state or disease conditions. Thus, it is paramount to select an appropriate single‐cell protocol for each individual study. The choice depends on the specific biological questions being addressed, and is influenced by several factors, including the depth of gene information needed, the number of cells to profile, and cost.^4^, ^41^ Protocols that sequence full‐length transcripts capture more comprehensive transcriptomes, including highly variable genes like TCRs. Such approaches facilitate the in‐depth functional interpretation of particular cell types (e.g., malignant cells and tumor‐infiltrating T cells), alternative splicing dynamics, and somatic mutation patterns.^42^ In contrast, massively parallel 3′ sequencing protocols reduce costs and raise throughput, albeit at the expense of sequencing depth. These approaches facilitate broad surveys of cellular components from complex tissues.^43^, ^44^, ^45^ The combined utility of these platforms will allow for a wider variety of biological questions to be probed by this technology.

Despite the advances of scRNA‐seq technologies, substantial limitations and challenges remain. One molecular limitation is the lack of unbiased identification of noncoding RNAs. This is due to the nature of the sequencing strategy, which specifically targets polyadenylated mRNA transcripts. Another limitation is that scRNA‐seq only provides a snapshot of transcriptomic information, while the genomic, epigenetic, and proteomic components of the cells are not captured. This hinders comprehensive understanding of the molecular mechanisms of cellular processes. Additionally, compared to single‐cell DNA genomics, scRNA‐seq exhibits limited sensitivity and is, therefore insufficient to reconstruct the clonal evolution of tumor cells in the TME. This limitation has only been somewhat overcome by applying gene expression‐based estimation of genomic alterations to define malignant cells.^22^, ^26^ Finally, because of the tissue sampling and disassociation processes, scRNA‐seq cannot yet map expression data to a precise anatomical location or cytoarchitecture. However, emerging spatially resolved transcriptomic methods can link cellular localization to molecular typing in neuronal tissues, and we expect these methods to be applied to immunological settings.^46^ Thus, scRNA‐seq technology is beginning to overcome initial limitations, and future advancements may allow us to concurrently examine genomic, epigenomic, proteomic, and spatial information from single cells.

### 2.2 Lineage tracing by scRNA‐seq

Identifying the lineages and relationships between cell types will provide detailed insights into tissue development and homeostasis, as well as how the dysregulation of these pathways contributes to pathologies like cancer. Lineage reconstruction with scRNA‐seq data has its origins in embryonic development research, where scRNA‐seq has been used to infer the trajectories based on pseudotemporal ordering of sequenced cells according to their similarity in gene expression.^47^ Monocle and related algorithms have since been applied to reveal the relationships of different immune cells and their progenitors during hematopoiesis, infection, and tumorigenesis.^8^, ^47^, ^48^ Although these trajectory inferences can connect developmental pathways, the biological interpretation of these data is limited by the need for prior knowledge and the assumption that pseudotemporal ordering is largely based on similarity.^49^ In some cases, these inferences may reflect the continuum of cellular states, rather than real developmental relationships.

Another way to trace cellular lineages is the analysis of genetic marks or scars. Such molecular identifiers can be induced via techniques such as CRISPR recombination. This methodology has been used to mark embryos and the juvenile brain of zebrafish.^50^, ^51^ However, due to technical limitations such as low labeling efficiency, this approach is restricted to specific experimental conditions. Another way to use genetic scars as lineage tracing tags is to measure mitochondrial genome mutations from human scRNA‐seq or scATAC‐seq data.^52^ This approach consistently traced T cell clonal expansion in multiple sample types, but its ability to delineate dynamic relationships of different T cell subsets has not yet been determined. For the lineage tracing of immune cells in human tumors, a more feasible approach has been to assess the endogenous genetic scars that exist in lymphocytes. For T cell and B cells, germline DNA recombination results in a vast repertoire of gene sequences for TCRs and B cell receptors (BCRs; also known as immunoglobulin). The high diversity of these repertoires makes it unlikely that two unrelated cells would exhibit identical TCR or BCR sequences. Thus, TCR‐ or BCR‐based sequencing could be used to define the clonality and track the dynamic relationships of these lymphocytes.

**FIGURE 1:**
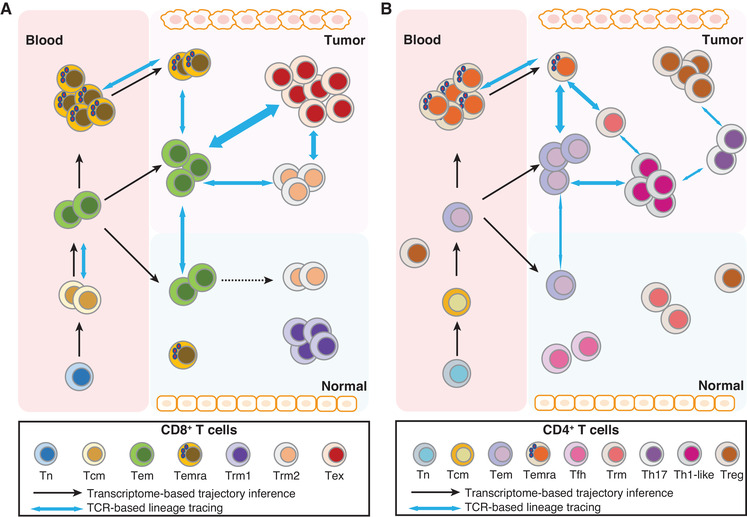
STARTRAC analyses define the dynamic status of T cell subsets in tumor. The dynamic status and relationship of CD8^+^ (A) and CD4^+^ (B) T cell subsets are inferred based on STARTRAC indices. Number of cells indicates the degree of clonal expansion. Black arrows show the potential developmental trajectory inferred by monocle. Blue arrows indicate the developmental relationship based on TCR sharing. The thickness of the arrows predicts the strength of the relationship

Previous studies based on bulk TCR‐α or TCR‐β sequencing revealed divergent T cell clonality in different tissues, as shown in an analysis of tumor‐infiltrating versus peripheral blood T cells from glioma patients.^53^ However, such bulk sequencing methods were not able to capture the underlying phenotypic differences among individual T cell clones. In contrast, the simultaneous detection of TCRs and other transcripts in single cells have started to unmask such differences. One pioneering study used quantitative RT‐PCRs to simultaneously detect TCRs and selected transcripts in single CD4^+^ T cells to reveal the clonal ancestry and differentiation of these T cells.^54^ This approach provided an avenue to link TCR identities and phenotypes, but was limited by its relatively low throughput. More recently, emerging scRNA‐seq technologies began to depict the clonality and developmental trajectories of individual T cells in the steady state, as well as in diseases such as infections and cancers, using integrated transcriptome and TCR analyses.^55^, ^56^, ^57^, ^58^, ^59^, ^60^ These studies greatly advanced our understanding of T cell dynamics in many tissues and disease states. However, further analysis of T cell dynamics across different tissues or subtypes required a more quantitative analytical platform. To this end, our group developed a new framework, called STARTRAC (single T cell analysis by RNA sequencing and TCR tracking).^61^ STARTRAC analyses provide additional insights into the properties of T cell subsets, especially in cancer (Fig. 1). For example, previous studies based on bulk TCR‐β sequencing or single‐cell inferred trajectories showed connections between effector, effector memory, and exhausted CD8 T cells in multiple cancer types, indicating a linear developmental differentiation of these T cells. However, our STARTRAC analyses revealed limited connections of effector and exhausted T cells in colorectal cancer (CRC), suggesting an underappreciated effector‐independent development of exhausted T cells.^61^ This observation was independently confirmed by a recent study of chronic infection, which showed that the effector versus exhausted fate decision occurred in a precursor subset, and not within the effector lineage.^62^ More detailed dissection of exhausted and other T cell lineages by STARTRAC and other single cell‐based analyses will be discussed in the following sections.

One limitation of the aforementioned approaches is the lack of directionality of the established trajectory. To overcome this obstacle, levels of unspliced precursor mRNA and spliced mRNA from scRNA‐seq data have been used to infer the future state of a cell on a timescale of hours.^63^ This framework, termed RNA velocity, has been successfully applied to reconstruct the neural crest lineage, mouse hippocampus development, human embryonic neurogenesis, and even a whole planarian.^64^ Since RNA metabolism is a relatively short‐term biological process, whether this approach can be applied to T cell lineage tracing in chronic conditions, such as cancer, remains to be seen.

## 3 TUMOR‐INFILTRATING T CELL CLUSTERS IDENTIFIED BY SCRNA‐SEQ

It has long been known that various leukocyte subsets, especially T cells, infiltrate human solid tumors. Studies of animal cancer models established that the immune system constantly surveils and eliminates primary transformed tumors, and that tumors evolve to establish an equilibrium and eventual escape from immune surveillance through cancer immunoediting.^65^ Animal models with deficient T cell functions were particularly susceptible to tumor outgrowth. Supporting these preclinical observations, it is also known that many human cancers with higher T cell infiltration have better prognostic outcomes.^66^ More importantly, T cell infiltration and activation status have been used as predictive markers to select for patients that respond to CPI treatment in many cancer types.^67^ Thus, a better understanding of T cell composition and regulatory pathways is key for the improvement of cancer immunotherapies. Previously, characterization of tumor‐infiltrating T cell subsets using immunohistochemistry and multicolor flow cytometry techniques demonstrated the presence of multiple T cell subsets in tumors. However, it was only with the recent advancement of scRNA‐seq technology that researchers have had the opportunity to uncover detailed characteristics of tumor‐infiltrating T cell subsets in an unbiased manner (Table 1). Using this technology, heterogeneous T cell subsets have been identified in various types of human tumors. Whereas the T cell clusters identified in cancer patient blood are largely consistent across different tumor types and are similar to those found in healthy donor peripheral blood mononuclear cells, tumor‐infiltrating T cell clusters defined by scRNA‐seq analysis vary depending on tumor origin, type, location, and disease stage. Nevertheless, several major intratumoral CD4^+^ and CD8^+^ T cell populations are identified across different tumors, including exhausted T (T_exh_) cells, effector memory T (T_em_) cells, effector or effector memory re‐expressing CD45RA T (T_eff_/T_emra_) cells, tissue‐resident is fine memory T (T_rm_) cells, and regulatory (Treg) cells. Herein, we will focus our discussion on up‐to‐date key findings of their properties and their role in antitumor immunity.

### 3.1 Exhausted CD8^+^ T cells in tumors

#### 3.1.1 Overview

The majority of intratumoral CD8^+^ T cells are functionally impaired and stably express inhibitory co‐receptors such as PD‐1, Tim‐3, and LAG3.^68^, ^69^, ^70^ Interestingly, these receptors are up‐regulated upon T cell activation and function to repress excessive T cell proliferation and effector functions. Stable expression of these receptors has since come to define a distinct lineage of dysfunctional T cells called exhausted CD8^+^ T cells (T_exh_ cells).^71^, ^72^, ^73^ The concept of T cell exhaustion originated in the 1960s but detailed examination of the lineage accelerated in the 1990s and 2000s with the implementation of mouse models of chronic infection, especially lymphocytic choriomeningitis virus (LCMV).^74^, ^75^ These cells are associated with a unique developmental pathway that arises due to repeated antigen exposure during chronic viral infection or cancer. Exhaustion is theorized to be a mechanism of avoiding autoreactivity and immunopathology while limiting tumor growth or chronic infection. T_exh_ cell properties include progressive hyporesponsiveness to TCR or cytokine stimulation, inability to form memory T cell pools, and a reprogrammed metabolic and epigenetic circuitry.^76^ Exhaustion is separated both phenotypically and developmentally from 2 other dysfunctional T cell fates, senescence, and anergy, with the primary difference being that T_exh_ cells have previously undergone initial activation.^71^ Blockade of PD‐1, perhaps the most well characterized inhibitory co‐receptor, can enhance the function and block the terminal differentiation of these cells to control chronic viral infection.^62^, ^77^ And in cancer settings, PD‐1 expressing intratumoral T lymphocytes are a predictive biomarker of cancer patients who may benefit from CPI treatments. Many studies have suggested that one of the major mechanisms of anti‐PD‐1 therapies is to revert the dysfunctional phenotypes of tumor‐infiltrating exhausted CD8^+^ T cells and enable them to better kill and control cancer cells.^67^ More recently, single cell‐based transcriptomics has been applied to dissect this surprisingly heterogeneous lineage in even greater detail, with a major goal being to decipher just how CPI treatments manipulate intratumoral CD8^+^ T cell lineages. Below, we discuss 5 areas of T_exh_ cell biology where single‐cell technologies have been particularly useful in furthering our understanding: molecular markers, properties, heterogeneity, developmental trajectory, and reactivity.

#### 3.1.2 Biomarker genes for T_exh_ cells

A major limitation within T_exh_ cell biology has been the lack of specific molecular identifiers of these cells. The inhibitory receptors traditionally used to identify the T_exh_ subset are broadly induced by TCR signaling on CD8^+^ T cells, and some are constitutively expressed on NK cells.^78^ Similarly, the reported transcription factors governing T_exh_ cells, including NFAT, Eomes, and TCF1, are also shared with other lymphocyte populations. The depth and resolution provided by single‐cell technologies repesent a powerful tool to identify more specific and actionable markers of exhaustion. Indeed, several new candidate genes have come out of such studies. For example, in a study of T_exh_ cells in liver cancer and melanoma,^59^, ^79^ novel markers such as *MYO7A, WARS, CXCL13, TOX, LAYN, PHLDA1*, and *SNAP47* were identified alongside well‐known T_exh_ genes such as *HAVCR2, PDCD1, ENTPD1 (*CD39*), CTLA4, TIGIT, TNFRSF9*, and *CD27*. Some of these newly identified marker genes were further shown to regulate the function or the development of CD8^+^ T_exh_ cells. For example, overexpression of LAYN inhibited effector functions of CD8^+^ T cells in in vitro systems.^59^ CD39, which had been initially identified as a specific T_exh_ cell marker during chronic infection^80^ was found to mark T_exh_ cells in several cancers. CD39 has since become a relevant cancer immunotherapeutic candidate.^16^, ^81^, ^82^ Finally, beyond transcript‐level markers of exhaustion, it has been argued that an epigenetic signature may be the most robust marker of exhaustion.^83^, ^84^ It has also been theorized that the epigenetic state of exhaustion would hinder any attempt to invigorate T_exh_ cells with lasting effect and therefore, reversing T_exh_ epigenetics would be key to any therapy targeting these cells. To that end, TOX, which is preferentially expressed in T_exh_ cells compared to other intratumoral subsets, was recently identified as a key mediator of chromatin remodeling and transcription in T_exh_ cells.^85^, ^86^, ^87^, ^88^, ^89^ TOX was shown to enhance T_exh_ cell survival and inhibitory receptor expression, although it does not alter the effector functions of T_exh_ cells. TOX is already known to play an important role in the development of several homeostatic leukocyte populations, but it is an intriguing exhaustion marker in cancer context given its pleiotropic effects within tumor T_exh_ cells.^90^

#### 3.1.3 In vivo phenotypes of T_exh_ cells

In addition to overexpressing various inhibitory cell surface markers, tumor‐infiltrating CD8^+^ T_exh_ cells have been defined by their inferior ability to respond to antigen stimulation, resulting in reduced effector functions, such as cytokine secretion, and lower proliferative potential. Most of these phenotypes were observed from ex vivo analysis of TILs isolated from human and mouse tumors.^91^, ^92^, ^93^ T_exh_ cells are not completely inert but maintain a suboptimal functionality that limits viral replication or tumor progression.^73^, ^94^, ^95^ Most importantly, blocking CPI pathways such as PD‐1 and Tim3 can invigorate these cells to control viral infections or eradicate tumors. However, the exact status of these cells inside tumors remained elusive until the recent emergence of scRNA‐seq. Based on TCR clonality analyses, such as STARTRAC, and combined transcriptome analyses, a surprising finding was that T_exh_ cells were the most clonally expanded and proliferative (KI67^high^) T cell subset in multiple cancer types. In addition, these cells highly expressed various effector molecules such as IFN‐γ and Granzyme B, despite their expression of inhibitory receptors. These data suggest that tumor‐infiltrating CD8^+^ T_exh_ cells are genuinely and constantly activated, probably through TCR engagement with tumor‐associated antigens presented by class I MHC. This would imply that their “exhaustion” state may in fact be normal T cell signaling in response to continuous antigen exposure within the highly immunosuppressive TME, providing a rationale for how CPI treatments reduce inhibitory signaling enough to reinvigorate T_exh_ cells.^94^, ^95^, ^96^ This premise is consistent with the finding that PD‐1^+^ intratumoral CD8^+^ T cells are the predominant tumor reactive CD8^+^ T cell clones.^97^ Relatedly, STARTRAC analysis showed that T_exh_ cells were less mobile compared to other T cell subsets, especially T_eff_ cells, and that these cells highly express some cell surface markers associated with T_rm_ cells, such as *ITGAE*.^61^ This implies a closer relationship of the T_exh_ lineage to memory T cell subsets than was previously appreciated.

#### 3.1.4 Heterogeneity of tumor‐infiltrating T_exh_ cells

Several studies have shown that T_exh_ cells can be further subdivided into distinct populations: early T_exh_ cells and terminal T_exh_ cells.^94^, ^98^, ^99^, ^100^ Early T_exh_ cells possess a stem‐like and memory‐like phenotype, express lower levels of effector transcripts, and possess a proliferative capacity that can seed the terminal T_exh_ cell population independent of other CD8 subsets. By contrast, terminal T_exh_ cells do not exhibit multipotency or memory‐like abilities, express high levels of effector transcripts, and are less proliferative. Interestingly, TOX expression and function provide a link between the T_exh_ subsets.^85^, ^86^, ^87^, ^88^, ^89^ In preclinical models and patient tumor biopsies, TOX expression is up‐regulated in early T_exh_ cells, where it establishes epigenetic signatures required for cell persistence. TOX expression is sustained in terminal T_exh_ cells, and genetic deletion of TOX in T cells results in reduced overall numbers of T_exh_ cells. Therefore, early and terminal T_exh_ cell subsets, which exhibit notable phenotypic and transcriptional divergence, are linked early in their developmental trajectory by epigenetic reorganization. Going forward, it will be important to determine the broad functionality of TOX‐dependent reprogramming in human intratumoral T_exh_ cells.

Given the abundance of T_exh_ cells in many tumor types, as well as mounting evidence of their role in immunotherapy responses, it is essential to better understand the molecular dynamics of this heterogeneous subset. Early T_exh_ cells express several markers, including CXCR5 and TCF1, that are not associated with T_eff_ cells. Subsequent single cell‐based analyses have since offered contrasting conclusions on precisely when and where TCF1 helps to establish T_exh_ cells. In cancers, these cells were first identified in NSCLC by high‐dimensional FACS analysis, and they expressed other signature genes shared by follicular helper T (T_FH_) cells in addition to TCF1.^101^ In another study of melanoma, TCF1 was overrepresented in a human effector/memory‐like tumor‐infiltrating T cell population that was predictive of checkpoint blockade responsiveness.^81^ Expression of TCF1 was, in fact, a marker used to separate T_eff_/T_mem_ cells from T_exh_ cells. However, in other recent studies, trajectory analyses of CD8^+^ T cells showed that TCF1 was both a marker and driver of early T_exh_ lineage establishment.^98^, ^100^ In one such study, done in mice chronically infected with LCMV, TCF1 specifically inhibited effector programs and promoted the establishment of early T_exh_ cells.^62^ Relatedly, another study of melanoma TILs showed TCF1 to be a marker of a highly replicative, transitional CD8^+^ population that resembles early T_exh_ cells.^102^ Notably, TCF1, a transcription factor, is also highly expressed in naïve T cells and regulates memory T (T_mem_) cell formation.^103^ Thus, it is becoming apparent that, although its precise expression pattern may be context‐dependent, TCF1 can promote memory functionalities broadly within activated CD8^+^ T cells. TCF1 may help multiple lineages of memory and memory‐like CD8^+^ T cells to maintain stem‐like abilities, including self‐renewal, proliferative capacity, and differentiation into terminal subsets. In several scRNA‐seq studies with human tumors, the early T_exh_ cells were classified as T_em_ populations because they share markers previously used to define effector memory cells.^59^, ^60^, ^61^ STARTRAC analysis revealed that some T_em_ cells are developmentally connected to T_exh_ cells and are potential precursors of the T_exh_ lineage (Fig. 1). Interestingly, we found that, at least in CRC samples, both TCF1^+^ and TCF1^−^ cells are embedded in the T_em_ cell population, and both populations are developmentally connected with T_exh_ cells^61^ (unpublished observation), suggesting that multiple developmental pathways can establish intratumoral T_exh_ cells.

Although CD8^+^ FOXP3^+^ Tregs have been observed in CRC and pancreatic cancer patients by flow cytometry,^104^ scRNA‐seq analyses have also identified these cells in various cancer types including HCC, lung cancer, and CRC.^59^, ^60^, ^61^ More importantly TCR lineage tracing studies revealed that some of these T cells shared TCRs with CD8^+^FOXP3^−^ T_exh_ cells, indicating a possible conversion between CD8^+^FOXP3^−^ T_exh_ cells and FOXP3^+^ T cells. Another study identified a subpopulation of cells that express both hallmarks of Treg cells (e.g., *FOXP3* and *CTLA4*) and cytotoxic molecules (e.g., *PRF1* and *NKG7*) within the exhausted CD8^+^ T cell subtype.^59^ Such analyses further the hypothesis that T_exh_ cells share and acquire a transcriptomic program with Treg cells. It also remains to be studied if these cells can exert suppressive functions like bona fide CD4^+^ Treg cells.

#### 3.1.5 Development of the T_exh_ lineage

The developmental pathway leading to terminally differentiated T_exh_ cells remains incompletely understood. Initial experiments led to the hypothesis that T_exh_ cells derived from T_eff_ cell precursors in a linear and progressive fashion during chronic antigen exposure.^105^ This trajectory was contrasted with the differentiation T_eff_ to T_mem_ cell transition that occurs after acute antigen exposure. Importantly, studies in animal models suggested the development of tumor‐specific CD8^+^ T_exh_ cells was a dynamic process dependent on continuous antigen exposure during early tumorigenesis.^92^ More recent work utilizing single cell transcriptome‐level analyses, has indicated that T_exh_ cell development may be partially or completely independent of the cytotoxic T_eff_ cell lineage and may exhibit more characteristics of memory formation than previously appreciated.

Gene expression and TCR‐indexing analyses have shown that a T_em_ cell population may be a transitional population preceding the lineage fate of T_eff_ and T_exh_ cells in tumors.^59^, ^60^, ^61^ Analyses of TIL populations from 3 types of cancer show that a CD8^+^GZMK^+^ T cell population exhibits characteristics of T_em_ cells and appears to share a lineage with both T_exh_ and T_eff_ cells.^61^ Previous studies based on bulk TCR‐β sequencing or single‐cell inferred trajectories showed connections between T_eff_, T_em_/T_rm_, and T_exh_ cells in multiple cancer types, indicating a linear developmental differentiation of these T cells. However, STARTRAC analysis revealed limited connections between T_eff_ and T_exh_ cells in CRC.^61^ Further analysis of the clonotypes of GZMK^+^ T_em_ cells in CRC revealed that the subpopulation of these cells shared TCRs with effector T cells were mutually exclusive with those shared TCRs with exhausted T cells, indicating a TCR‐based fate decision. It is unclear whether such a mutually exclusive pattern can be generalized to different cancer types.

As discussed earlier, studies of preclinical models and human tumors suggested that TCF1^+^ T_exh_ precursor or stem‐like cells can develop into T_exh_ cells. However, lineage tracing analysis with STARTRAC reveals both TCF1^+^ and TCF1^−^ subpopulations reside in the T_em_ clusters and are comparably connected with T_exh_ cells developmentally^61^ (unpublished observation). Also of note, analysis of NSCLC indicates that T_rm_ cells share a direct link to the T_exh_ cell lineage, indicating that the TME may broadly divert T_em_ cell recall responses to induce T_exh_ cells.^60^ Altogether, these data suggested potentially diverse origins of tumor‐infiltrating CD8^+^ T_exh_ cells.

#### 3.1.6 Reactivity of tumor T_exh_ cells

T_exh_ cells have been shown in multiple settings to actively contribute to immune responses.^77^, ^85^ However, it remains unclear if they can be reinvigorated in a clinical setting to provide long‐term cures against chronic infections or cancers. It is further unknown if broad reactivation of T_exh_ cells could elicit cures without immunopathological side‐effects. And in the context of PD‐1/PDL1 blockade, the relevant CD8^+^ population being activated (or reactivated) in clinical responders has not been convincingly identified. To address these questions, several groups have applied single cell analyses to patient tumor samples to identify highly active and reactive CD8^+^ T cells. A study of CRC indicates that T_exh_ cells exhibit high TCR clonality and are by far the most actively proliferating cell type in these tumors, suggesting that T_exh_ cells possess at least some responsiveness that could be further manipulated for therapeutic benefit.^61^ However, studies based on mouse models suggest that not all T_exh_ cells are reactive. Terminal T_exh_ cells with high PD‐1 expression could not be reinvigorated, but rather T_exh_ precursor/stem‐like cells with low or intermediate expression of PD‐1 are reactive to CPI treatment.^94^, ^95^

Analyses of melanomas have been particularly insightful, even if the studies’ conclusions have important contrasts. One such study highlighted the heterogeneity and proliferative capacity of the T_exh_ compartment, going so far as to correlate T_exh_ cell abundance with reactivity to autologous tumor cells ex vivo.^102^ In agreement with this, analysis of circulating CD8^+^ T cells following checkpoint blockade found that the most proliferative cells had an exhausted phenotype.^106^ However, a separate study showed that responsiveness to checkpoint blockade was predicated on a low ratio of T_exh_ cells within melanoma lesions.^81^ Another recent study tracked site‐matched melanoma lesions before and after checkpoint blockade to better understand the origins and phenotypes of tumor‐reactive CD8^+^ clonotypes.^82^ Surprisingly, while checkpoint blockade preferentially expanded T_exh_‐like cells, those clones were mostly not matched in pretreatment infiltrates. This suggests that checkpoint blockade elicits clonal replacement of CD8^+^ T cells, and the new infiltrates then become exhausted themselves. Combined, these studies show that there are highly heterogeneous T_exh_ and T_eff/mem_ populations in tumors, all of which remain potential targets of immunotherapy. More detailed studies of tumor‐reactive CD8^+^ T cells, and large‐scale identification of their tumor‐associated Ags, is needed to determine when and where each cell population could be manipulated.

### 3.2 Characterization of Treg cells by scRNA‐seq

Treg cells are a subset of CD4^+^ T cells that play a central role in maintaining immune homeostasis. Treg cells function by suppressing responses of other immune cells thereby maintaining peripheral tolerance and limiting host damage that can result from an exaggerated or unchecked immune response.^107^ Importance of Treg cells is well recognized both in context of autoimmunity, where they can be protective, as well as in cancer, wherein their presence impedes tumor clearance. Transcription factor Foxp3 plays a major role in controlling all facets of Treg cell biology ranging from lineage specification and stability to function with a substantial proportion of Treg cell transcriptional signature being Foxp3 dependent. While Foxp3 expression is generally restricted to Treg cells, it can be transiently expressed upon activation in conventional T cells in human. Furthermore, Treg cells can lose Foxp3 expression under certain inflammatory conditions and convert into pathogenic effector cells.

Treg cells have been reported to exhibit considerable heterogeneity based on developmental origin (thymic derived vs. peripherally induced), expression of activation markers (such as CD62L and CD44 that mark central and effector Treg subsets),^108^ expression of distinct transcription factors (Tbet, Gata3, RORγt, and Bcl6 that corresponds to functional Treg subsets that are specialized toward suppression of particular T helper responses)^109^ presence in certain anatomical locations such as skin, intestine, and fat that can imprint tissue specific features (like expression of genes such as *PPARγ* and *IL‐33R*)^110^ as well as mechanisms utilized for suppression of other immune cells (sink for growth factors like IL‐2, expression of suppressive molecules such as CTLA4 and granzyme mediated direct cytotoxicity).^111^ scRNA‐seq therefore provides a useful approach to understand the diversity in Treg cell origin and function.

#### 3.2.1 Regulatory T cells in normal tissues and inflammatory diseases

ScRNA‐seq analysis of splenic CD4^+^ conventional T cells and Treg cells sorted from unperturbed mice has revealed that they mostly cluster separately with a small degree of overlap.^56^, ^112^ Profiling of Treg cells by scRNA‐seq in non‐lymphoid tissues such as skin and colon has identified some interesting features.^113^ As compared to lymphoid tissues, Treg cells in these two compartments showed substantial enrichment of genes that are part of the TNFR‐NF‐kB signaling axis such as *TNFRSF4*, *TNFRSF9*, *TNFRSF18*, and *Pim1/2*, suggesting that the TNFR pathway might play a role in modulating Treg cell homeostasis and function in these tissues.

Analysis of two discrete thymic Treg cell precursors (CD25^+^Foxp3^−^ and CD25^−^Foxp3^lo^) by scRNA‐seq has shown that they have unique transcriptional signatures that were reflective of distinct modes of differentiation.^114^ The CD25^+^ precursors were more enriched in genes associated with stronger TCR signaling while the Foxp3^lo^ subset had increased expression of adapter genes that could enhance signaling via TNFRs and TCR. These differences in progenitor cells in turn resulted in production of Treg cells that were qualitatively different as measured by their ability to suppress experimental autoimmune encephalomyelitis.

Treg cells play a prominent role in suppressing autoimmunity and scRNA‐seq analysis of biopsy samples from animal models and inflamed tissues of patients suffering from autoimmune disorders has provided unique insights into their phenotype and function. During ulcerative colitis (UC), a subtype of inflammatory bowel disease, an enrichment of Treg cells has been previously reported in the colonic mucosa. In this setting, scRNA‐seq has uncovered that TNF expression, which is one of the prominent pathogenic drivers in this disease, shifts dramatically toward Treg cells.^115^ In tissues from healthy patients and non‐inflamed tissue from UC patients, TNF is mostly expressed by activated CD4^+^ T cells and tissue resident CD8^+^ T cells, but in inflamed tissue, Treg cells are one of the major sources of TNF suggesting that they might have converted into effector‐like cells. However, these Treg cells maintain expression of characteristic genes (*FOXP3*, *CTLA4*, *IL10*), so more work is needed to identify whether they promote pathogenesis or resistance to anti‐TNF therapy. Furthermore, up‐regulation of *IL18* by enterocytes correlates with this increased presence of Treg cells (that express IL18R1) in inflamed colonic mucosa indicating that Treg cell recruitment may be regulated by the epithelial cells during UC.

#### 3.2.2 Regulatory T cells in cancer

Beyond providing a better understanding of Treg cell diversity during homeostasis, scRNA‐seq technology has helped elucidate their role during cancer. Relevance of Treg cells in cancer is highlighted by the fact that their increased presence often predicts poor prognosis and several therapeutic strategies designed to deplete them show efficacy. Analysis of infiltrating cells isolated from several different human tumors (liver, lung, breast, skin, and colon) by scRNA‐seq has identified a Treg cell gene signature that is distinct from normal tissue‐associated Treg cells.^43^, ^59^, ^60^, ^61^, ^79^ Comparing all these studies has yielded a common set of genes such as *CTLA4*, *TNFRSF4*, *TNFRSF18*, *TIGIT*, *ICOS*, and *CCR8* whose expression is higher in tumor‐associated Treg cells as compared to Treg cells from other tissues. Along with these genes whose function in Treg cells have previously been characterized, several other genes such as *LAYN*, *CD177*, *IGFLR1*, and *IL1R2* that are not well studied are also up‐regulated in tumor Treg cells. A more detailed examination has revealed patterns of heterogenous gene expression in tumor Treg cells. Expression of *TNFRSF9* (encoding CD137; 4‐1BB) demonstrated a bimodal distribution in tumor Treg cells and as *TNFRSF9* is known to be uniquely up‐regulated in Treg cells upon TCR stimulation^116^, this subset might represent Ag‐activated Treg cells. Genes highly enriched in CD137^hi^ Treg cells, as compared to tumor Treg gene signature, corelated with worse patient prognosis in the TCGA lung adenocarcinoma dataset suggesting that CD137^hi^ Treg cells correspond to suppressive tumor Treg cells.^60^

Co‐variance in gene expression has also been described in tumor Treg cells with co‐expression of genes such as *CTLA4*, *TNFRSF18*, and *TIGIT* in certain Treg cell clusters with mutually exclusive expression of these genes in other Treg cell clusters indicating that they may occupy distinct spatial or functional niches.^43^ Interestingly, a small subset of genes enriched in tumor Treg cells such as *CTLA4*, *TIGIT*, *TNFRSF9*, *CD27*, and *LAYN* are also highly expressed by exhausted tumor‐infiltrating CD8^+^ T cells reflecting a shared program of activation and exhaustion in these cells. Along with CD8^+^ T cells, tumor‐infiltrating Treg cells are among the most highly clonally expanded population suggesting that they undergo local expansion after recognizing tumor‐associated Ags. Lineage tracking analysis using TCR repertoire has revealed that the source of these tumor‐infiltrating Treg cells is mostly recruitment from other lymphoid tissues with migration from adjacent tissues and conversion of CD4^+^ T cells to induced Treg cells providing only a minor component. Based on TCR sharing analysis, the induced Treg (iTreg) cells could be developmentally linked to either Th1‐like (*BHLHE40^+^CXCL13^+^*) or Th17 cells with *BACH2* being selectively expressed in Th1‐like iTreg cells and *RORC* and *SATB1* preferentially enriched in Th17 linked iTreg cells suggesting that different Treg cells subsets are present with in the TME.^61^ Although gene expression profile of tumor Treg cells and their derivation from lymphoid tissues has previously been reported using bulk RNA‐seq,^117^, ^118^ scRNA‐seq has provided a clearer picture of tumor Treg cells and identified diverse subsets whose function is not yet well defined.

Overall, scRNA‐seq has been very informative in providing a better understanding of Treg cell diversity during various aspects of their development, tissue residence and function during inflammation and cancer.

### 3.3 Other memory T cell subsets in tumor

Besides tumor‐enriched T_exh_ and Treg cells, scRNA‐seq analysis also identified additional T cell clusters that showed various cross‐tissue distribution between tumor and blood and/or normal tissues. These include naïve T cell (T_n_), central memory T cell (T_cm_), T_em_, and T_emra_ or T_eff_ for both CD4^+^ and CD8^+^ T cells (Table 2). Within memory CD4^+^ T cells, different T helper (Th) subsets, including Th1, Th2, Th17, and T follicular helper (T_FH_) can also be identified. The signature genes identified by scRNA‐seq for these T cell clusters are largely consistent with previous studies that utilized microarray or bulk RNA‐seq and show parallel patterns in human CD4^+^ and CD8^+^ T cell lineages.^119^, ^120^ CyTOF analysis at protein level has confirmed the presence of these T cell subsets in tumor.^13^, ^14^ Previous studies with combined phenotypic, functional, epigenetic, and gene expression properties of these T cell subsets suggest a linear T cell progression model (T_n_‐T_cm_‐T_em_‐T_emra_) along these T cell clusters at the quiescent state.^121^, ^122^, ^123^, ^124^, ^125^ Similarly, inferred developmental trajectory of these T cell clusters in scRNA‐seq datasets based on either transcriptome or incorporated with TCRs also exhibited a continuous structure of these cells with T_em_ tending to be the intermediate cells.^59^, ^60^, ^61^ However, scRNA‐seq revealed that these different subsets inside tumors may not display as discrete clusters, instead, in vivo isolated T cells demonstrate broad continuum of activation and differentiation transcriptome spectrums surrounding the core subset defining gene signatures, which is likely dictated by both TCR/Ag specificity and environmental factors.^43^, ^59^, ^60^, ^61^, ^79^

**TABLE 1:** Summary of single cell studies of human tumor‐infiltrating immune cells/T cells

Tissues	Single‐cell technologies	Platforms	Targeting cells	References
Melanoma	scRNA‐seq	Smart‐seq2	Pan‐immune and non‐immune cells	79
Renal cancer	Mass cytometry	CyTOF	Innate immune cells	14
Breast cancer	scRNA‐seq	Smart‐seq2	Pan‐immune and non‐immune cells	127
Lung cancer	Mass cytometry	CyTOF	Innate immune cells	13
Head and neck cancer	scRNA‐seq	Smart‐seq2	Pan‐immune and non‐immune cells	130
Liver cancer	scRNA‐seq	Smart‐seq2	T cells	59
Breast cancer	scRNA‐seq	InDrop & 10x Genomics	Pan‐immune cells	43
Lung cancer	scRNA‐seq	Smart‐seq2	T cells	60
Renal cancer	scRNA‐seq	10x Genomics	Pan‐immune and non‐immune cells	45
Lung cancer Colorectal cancer	Mass cytometry	CyTOF	CD8^+^ T cells	12
Breast cancer	scRNA‐seq	10x Genomics	Pan‐immune cells	128
Lung cancer	scRNA‐seq	10x Genomics	Pan‐immune and stroma cells	44
Colorectal cancer	scRNA‐seq	Smart‐seq2	T cells	61
Melanoma	scRNA‐seq	MARS‐seq	T cells	102
Melanoma Therapy	scRNA‐seq	Smart‐seq2	Pan‐immune and non‐immune cells	169
Melanoma Therapy	scRNA‐seq	Smart‐seq2	Pan‐immune cells	81
Breast cancer	Mass cytometry	CyTOF	Pan‐immune and non‐immune cells	15

Abbreviations: CyTOF, cytometry by time‐of‐flight.

**TABLE 2:** CD8^+^ T cell subsets identified based on the integrated single‐cell studies

T cell subset/state^a^	Gene signature	Annotation	Tumor type	Tissue enrichment^b^	Clonality	Mobility	Transition	Other tumor^c^	Functional interpretation
LEF1^+^ Tn	TCF7, SELL, LEF1, CCR7	Naive	CRC HCC NSCLC	Blood^b^	Low	Low	Low	Melanoma (Triosh)	
GPR183+ Tcm	GZMK, low GZMA/PRF1/NKG7/TCF7/EOMES/GPG183	Central memory/naïve‐like/memory	CRC HCC NSCLC	Blood^b^	Intermediate	Low	Intermediate	Lung cancer (Clarke) Melanoma (Li; Sade‐Feldman)	Correlated with response to ICB in melanoma (Sade‐Feldman)
CX3CR1+ Temra	GZMA, GNLY, PRF1, GZMB, NKG7, TBX21, ZEB2, HOPX	Effector memory recently activated /Effector/Cytotoxic	CRC HCC NSCLC	Blood^b^	High	High	High	Melanoma (Triosh, Li; Sade‐Feldman) Breast cancer (Savas) HNSCC (Puram)	
GZMK+ Tem	GZMK, EOMES, TOX, others similar to Tcm	Effector memory/Transitional	CRC HCC NSCLC	Tumor, Normal	Intermediate	Intermediate	High	Melanoma (Li; Sade‐Feldman) Breast cancer (Azizi, Savas)	
ZNF683+ Trm	IL2, ZNF683, HOPX, ID2, low effector molecules	Tissue‐resident memory/Pre‐exhausted	NSCLC	Tumor, Normal	Intermediate	Intermediate	Intermediate		
LAYN+ Tex	LAG3, TIGIT, PDCD1, HAVCR2, CTLA4	Exhausted/Dysfunctional	CRC HCC NSCLC	Tumor	High	Low	Intermediate	Melanoma (Triosh, Li; Sade‐Feldman) Breast cancer (Azizi, Savas) HNSCC 9Puram), Lung cancer (Clarke)	Highly proliferative (Melanoma, breast cancer, CRC, lung cancer), correlated with response to ICB in melanoma (Sade‐Feldman), correlated with tumor‐reactivity (Li)
CD6+ Trm	XCL1, XCL2, MYADM, CD6	Tissue‐resident memory	CRC	Normal	Intermediate	Intermediate	Intermediate		
CD160+ IEL	KLRC1/2/3, IKZF2, NR4A3, CD69, NR4A1/2, CD160	Intraepithelial lymphocyte	CRC	Normal	Intermediate	Low	Low		
SLC4A10+ MAIT	SLC4A10, ZBTB16	Mucosal associated invariant	CRC HCC NSCLC	Tumor, Normal, Blood	Intermediate	Intermediate	Low		

a The T cell subsets are summarized based on the integrated analysis of single cell transcriptome of T cells isolated from CRC, HCC, and NSCLC. All of these T cell transcriptomes were generated from the same scRNA‐seq platform (Smart‐seq2), and T cell subsets were obtained by re‐clustering analysis of these three datasets.

b The tissue enrichment of each T cell subset is summarized based on the calculation of their distribution in blood, tumor and adjacent normal tissue. For T cell subsets enriched in blood, such as *GPR183*+ Tcm, these cells were also identified in the tumor with less abundance.

c The T cell subsets identified by the above integrated analysis were aligned with other T cells based on the similar signature genes. Other tumor types/datasets with similar T cell subsets are listed here. The different annotations of these T cell subsets are also listed in the column “Annotation.”

#### 3.3.1 Tumor T cell clusters shared with blood and normal tissues

Among these T cell clusters, T_n_ and T_cm_ subpopulations are mainly found in patient blood but very rarely in tumor, consistent with their biological property as circulating T cells that traffic between blood and secondary lymphoid organs.^59^, ^60^, ^61^, ^79^, ^102^, ^126^, ^127^ Both T_em_ and T_emra_ cells are present in blood, normal tissue, and tumor, with T_em_ cells being relatively abundant in tumor as well as normal tissue whereas T_emra_ cells are predominantly observed in blood.^59^, ^60^, ^61^ T_emra_ cluster expresses high level of *S1PR5* and distinct cell adhesion molecules and chemokine receptors than other T cell clusters in tumor, rendering these cells high mobility.^60^, ^61^ We and others have also identified T_rm_ and mucosal‐associated invariant T (MAIT) cells in tumor.^59^, ^61^, ^128^ The importance and function of MAIT cells in cancer immunity has been reviewed recently and will not be discussed here.^129^

#### 3.3.2 Tumor‐infiltrating T_emra_ cells

Both CD4^+^ and CD8^+^ T_emra_ clusters represent a small proportion of tumor‐infiltrating T cells in various cancer types.^59^, ^60^, ^61^ Similar CD4^+^ and CD8^+^ clusters expressing high cytotoxic signature genes (e.g., *GZMA, GZMB, PRF1, GNLY, NKG7*) but low or no exhaustion signatures (*PDCD1*, *LAG3*, and *CTLA4*) are also reported in scRNA‐seq studies for melanoma^79^ and head and neck cancer^130^, ^131^ (Table 2). The less abundance of T_emra_ cells in tumors based on scRNA‐seq analysis is consistent with previous observations by traditional flow cytometry analysis (identified as CD45RA^+^CCR7^−^ CD4^+^ or CD8^+^ T cells) in multiple cancer types.^131^, ^132^, ^133^, ^134^

T_emra_ cells in healthy donor PBMC are found more frequently in CD8^+^ compartments and represent Ag‐experienced terminally differentiated memory T cells that are capable of immediate cytokine production and cytotoxicity without proliferation.^135^, ^136^ CD4^+^ T_emra_ cells share similar phenotypes as CD8^+^ T_emra_ cells with drastic variability in their frequency between individuals.^137^ Both CD4^+^ and CD8^+^ T_emra_ cells have been implicated in protective immunity against pathogens and contain expanded viral‐specific clones.^135^, ^136^, ^137^ Similarly, both CD4^+^ and CD8^+^ T_emra_ populations in cancer patients are found to be clonally expanded.^59^, ^60^, ^61^ Although the functional role of CD4^+^ T_emra_ population in cancer is still unclear, a recent scRNA‐seq study revealed that in humans, these cells might have developed from the precursors that express IL‐7 receptor, and the TCRs from these cells were clonally expanded and recognized dengue virus if the donors had been previously infected, supporting their role in viral control.

It is still controversial whether CD8^+^ T_emra_ cells contain clonotypes that are specific for tumor Ags. Li et al. identified T_emra_‐like cluster in melanoma tumor with high expression of cytotoxic‐related genes (e.g., *FGFBP2, GZMH, KLF2*) and low expression of exhaustion signature genes. However, T cells from those tumors with high intensity of cytotoxic signature were associated with low tumor reactivity in an ex vivo assay, suggesting tumor Ag‐specific T cell clones were not enriched in such tumor‐infiltrating T_emra_ cluster.^102^ In our CRC scRNA‐seq study, we found that T_emra_ cells were highly clonally expanded.^61^ The STARTRAC‐expansion analysis in this study showed that the degree of clonal expansion of T_emra_ cells in CRC patients was as high as T_exh_ cells and higher than other memory T cell clusters. Among clonally expanded T_emra_ cells, nearly half of them shared TCRs with tumor T_em_ cells, whereas only a small fraction shared TCRs with blood T_em_ cells. By introducing another 2 indices, STARTRAC‐transition and STARTRAC‐migration, for the measurement of state transition and tissue migration of T cell clusters, respectively, this study also showed that T_emra_ exhibited significantly higher developmental connection with T_em_ than with other T cell clusters and had the highest capability to migrate.^61^ Together, these findings suggest that at least some T_emra_ cells may have differentiated from tumor‐specific memory T cells and these cells can circulate between peripheral blood and tumor. Indeed, blood T_emra_ cells have been demonstrated to contain tumor Ag‐specific TCR clonotypes in breast cancer patients.^138^, ^139^ Percentage of blood T_emra_ cells was significantly higher in those NSCLC patients that partially responded to anti‐PD1 (Nivolumab) treatment than in non‐responders at baseline.^140^ In contrast, high blood T_emra_ cells in melanoma patients were found to associate with worse outcome in these cancer patients,^141^ suggesting a context‐dependent effect of T_emra_ cells.

Nevertheless, NSCLC patients who responded to Nivolumab had increased frequency and activity of tumor Ag‐specific T_emra_ cells, suggesting the antitumor activity of these cells at least in some tumor types.^138^, ^140^ T_emra_ cells express low levels of T_exh_ signature genes (e.g., *PDCD1* and *CTLA4*) but high levels of NK‐associated receptors (e.g., *CD94/NKG2A, KIRs, LILRB1*, and *KLRG1*), indicating distinct functional regulatory pathways for T_emra_ cells. Recently, we have demonstrated that PD1 blockade and LILRB1 blockade can synergistically enhance CD8^+^ T cell activity and cytolytic function in vitro.^142^ Given the high potency of cytolytic activity of T_emra_ cells,^142^ understanding how to promote tumor‐specific T_em_ cell differentiation to T_emra_ and how to improve their antitumor activity will be beneficial for immunotherapy.

#### 3.3.3 Tumor‐infiltrating T_em_ cells

T_em_ cells are also Ag‐experienced T cells that can rapidly elicit effector responses during Ag re‐encounter.^143^ T_em_ cells have better survival and proliferation capability than T_emra_ cells.^136^, ^144^ These cells differentiate from naïve T cells during primary immune response to Ag, with a distinct phenotypic and functional profile that allows them to migrate to blood, secondary lymphoid organs, and tissues.^125^, ^145^ Prior gene expression profiling by microarray or bulk RNA‐seq studies of human memory T cell subsets as defined by cell surface markers CD45RA and CCR7 showed a continuous transcriptomic change for these memory T cell subpopulations.^119^, ^120^, ^146^ Recent scRNA‐seq studies provide additional granularity and unbiased analysis for tumor‐infiltrating memory T cell subsets in cancer patients.

Our scRNA‐seq data identified that both CD4^+^ and CD8^+^ T_em_ cells were characterized by high expression of GZMK and intermediate expression of PD1.^59^, ^60^, ^61^ Similar GZMK^+^ T cell cluster has also been identified in almost all tumor types based on different scRNA‐seq approaches (Table 2). Both pseudotime trajectory and TCR sharing analysis suggest T_em_ cluster as the center of developmental path with one end linked to T_n_‐T_cm_ and the other end linked to T_emra_ or T_exh_. However, as mentioned before, scRNA‐seq analysis in CRC revealed that T_em_ clones linked to T_emra_ and T_exh_ are mutually exclusive.^61^ Such diverge developmental pattern of T_emra_ and T_exh_ cells is also observed in other cancer types like melanoma.^102^ Diametric linkage of T_em_ with T_emra_ or T_exh_ in tumor suggests that tumor T_em_ cells may take distinct differentiation paths toward T_exh_ or T_emra_ and that TCR specificity may have a role to determine their developmental trajectories. However, it is unclear whether this observation can be applied broadly to other cancer types or situations. Recently, CXCR5^+^ T_em_‐like CD8^+^ T cells possessing stem‐like properties were identified in NSCLC tumor by CyTOF and scRNA‐seq analyses.^101^ These cells are precursors of T_exh_ cells that were initially identified during chronic viral infection and are the primary cell types responding to CPIs.^98^, ^100^ Upon ex vivo stimulation, these cells isolated from tumor proliferate and gradually acquire T_exh_‐like phenotype and property.^101^ As discussed above, we noticed that these CXCR5^+^/TCF1^+^ cells are embedded inside the T_em_ population and are equally connected with T_exh_ cells as those TCF1^−^ T_em_ cells in multiple cancers^59^, ^60^, ^61^ (unpublished observation). These data support the hierarchical differentiation of T_em_ cells to become T_exh_ cells in the context of TME.

It has been shown that T_em_ cells contain tumor‐specific subsets in cancer.^147^, ^148^ In agreement with these findings, we found that tumor T_em_ cells had significantly higher STARTRAC‐expansion index in CRC (Fig. 1), suggesting that these cells underwent clonal expansion in response to local tumor‐associated Ag stimulation.^61^ Given the emerging evidences of tumor‐reactivity of T_exh_ cells, high STARTRAC‐transition index between T_em_ and T_exh_ cells also indicated the presence of tumor‐specific clonotypes in the T_em_ cluster.^61^ Moreover, positive correlation of T_em_ gene signature with better outcome in cancer patients^60^ suggests potential beneficial effects of therapeutic strategies to expand and activate T_em_ cells in tumor. A previous study using flow cytometry showed that the most prominent response to anti‐PD1 treatment was the expansion of intratumoral CD8^+^ memory T cells.^149^ In line with this observation, a recent scRNA‐seq study showed that there were more expanded clones in memory CD8^+^ T cell cluster than newly emerged clones under anti‐PD‐1 treatment.^150^ Nevertheless, further investigation is needed to validate the tumor‐reactivity of these tumor T_em_ cells and explore the extrinsic and intrinsic factors that allow their discrimination from bystander T cells.

Intratumor CD4^+^ T_em_ cell cluster is highly complex. Beside the major GZMK^+^ T_em_ clusters, Th17 cells, T_FH_, and T follicular regulatory subsets have also been identified in CRC samples.^61^ Th17 cells are characterized by the marker genes such as *RORC* and *IL‐23R*. STARTRAC analysis has also revealed these intestine related Th17 cells are developmentally connected to Treg cells, supporting the conversion of these 2 cell types in this tissue. The majority of cells in CD4^+^ T_em_ cluster are IFN‐γ positive with high expression of *EOMES* and *RUNX3*, suggesting these cells may be the bona fide Th1 cells. Interestingly, in CRC, another IFN‐γ^+^ Th1‐like cluster was identified that also expressed higher *BHLHE40* than traditional CD4^+^ T_em_ cells.^61^ Intriguingly, microsatellite‐instable (MSI) CRC patients have higher BHLHE40^+^ Th1‐like CD4^+^ T cells than microsatellite‐stable (MSS) patients.^61^ Given previous observation of better responses in MSI CRC patients,^151^ the preferential enrichment of CD4^+^ Th1‐like T cells in these patients indicate a positive correlation of these T cells with response to anti‐PD1 treatment. Furthermore, IGFLR1 was found to be highly expressed on Th1‐like CD4^+^ T cells in CRC, which may represent a novel costimulatory pathway to enhance IFN‐γ production from these cells.^61^ Although previous studies based on bulk RNA detection inferred the association between increased Th1 signature and the MSI status in CRC patients,^152^ scRNA‐seq analysis further illustrated that only CXCL13^+^BHLHE40^+^ Th1‐like cluster but not the classical CD4^+^ T_em_ cluster in CRC was preferentially enriched in MSI patients and could be accountable for the favorable response of these patients to CPIs.

#### 3.3.4 Tumor‐infiltrating T_rm_ cells

Tissue‐resident memory T cells (T_rm_) are memory CD4^+^ or CD8^+^ T cells retained in peripheral tissues to patrol nonlymphoid organs for protection from pathogen infection.^153^ These cells can be found in normal tissues, expressing CD69 and CD103 and devoid of CCR7 or CD62L.^154^, ^155^ Several transcription factors are involved in T_rm_ generation and maintenance, including the up‐regulation of RUNX3, Blimp‐1, and Hobit (encoded by *ZNF683*), and down‐regulation of EOMES and KLF2.^156^, ^157^, ^158^, ^159^ Emerging evidence has shown that T_rm_ cells are important for antitumor immunity mainly based on the positive correlation of CD103^+^ T_rm_ level with favorable prognosis in cancer patients.^160^, ^161^, ^162^, ^163^, ^164^, ^165^, ^166^ Although CD103 is a hallmark of T_rm_ cells, especially for those of epithelial origin, liver‐resident T_rm_ cells are characterized by the up‐regulation of integrin LFA‐1 instead of CD103.^167^ Moreover, for CD103^+^ T cells, recent studies have also identified different phenotypic populations within these cells in the TME. One example is the scRNA‐seq analysis of T_rm_ cells in lung cancer. In this study, multiple clusters were identified from sorted CD103^+^ T_rm_ cells, including T_exh_‐like cluster and classical normal tissue T_rm_ cluster.^168^ Another example is that in breast cancer, tumor‐infiltrating CD103^+^ CD8^+^ T cells were also separated into 2 clusters by scRNA‐seq analysis, with 1 cluster expressing higher level of both effector molecules and inhibitory receptors (such as *PRF1* and *GZMB*; *HAVCR2* and *LAG3*) and the other cluster expressing genes related to cell proliferation (such as *CCNA2* and *TUBB*).^128^ Therefore, these studies suggest that different clusters of tumor‐infiltrating CD103^+^ CD8^+^ T cells may have distinct phenotypic and functional properties and scRNA‐seq technology provides granularity for us to better understand the heterogeneity of these cells in tumor.

The recent scRNA‐seq studies of T cells from blood, normal tissue, and tumor of HCC, NSCLC, and CRC patients comprehensively characterize diverse T cell clusters enriched in tissues and revealed the heterogeneity of T_rm_ cell subsets in different cancer types.^59^, ^60^, ^61^ First, multiple T cell clusters were identified in tumor and the adjacent normal tissue, including T_em_, T_exh_, and T_rm_ CD8^+^ T cell clusters, expressed T_rm_ signature genes as observed in aforementioned studies.^132^, ^168^ However, detailed gene expression analysis revealed distinct expression patterns of these CD8^+^ T cell clusters: T_em_ cluster expressed low‐level *ITGAE* (encoding for CD103) but high‐level *PDCD1*; T_rm_ exhibited the opposite pattern; and T_exh_ showed high‐level for both genes.^60^ Second, the classical T_rm_ cells were present primarily in normal tissue than in tumor. The proportion of intratumoral T_rm_ cells was different in distinct tumor types. For example, the integrated analyses of T cells in HCC, NSCLC, and CRC revealed that although *ZNF683* ^+^ CD8^+^ T_rm_ cells were present in these 3 cancer types, their frequency was significantly higher in NSCLC than in HCC and CRC, suggesting potential function of these T_rm_ cells in NSCLC immunity.^60^, ^61^ Moreover, the T_rm_ cluster in different tumor types displayed diverse gene expression properties, mainly associated with the tissue origin of a given tumor. As an example, CD160^+^ IEL cluster, as characterized by high expression of both NK and inhibitory receptors like *ENTPD1* (encoding CD39) was found in the adjacent normal tissue from CRC but not NSCLC or HCC patients.^61^

One critical question about T_rm_ is whether they have different differentiation paths in diverse tissues or tumors. Pseudotime‐based analysis showed that in NSCLC *ZNF683* ^+^ T_rm_ located more centrally as T_em_ in the trajectory plot, suggesting these cells were at “pre‐exhaustion” state.^60^ Moreover, these *ZNF683* ^+^ T_rm_ cells were also found to share TCRs with T_exh_ cells and to a less extent with T_em_ cells.^60^ In CRC, CD6^+^ T_rm_ cluster showed dominant linkage with T_em_ but limited linkage with T_exh_ clusters, while CD160^+^ IEL cells were barely linked to other T cells based on STARTRAC‐transition analysis, indicating the different origins of T_rm_ cells in CRC.^61^ Finally, STARTRAC based mobility analyses also revealed that T_em_ cells are more mobile than both T_rm_ subsets and T_exh_ cluster^61^ (Fig. 1). Together, these findings support previous observation that T_em_ can give rise to T_rm_ in some tissues,^154^ but the T_exh_ cells may be derived from T_em_ and/or T_rm_ compartments in tumor. It will be of great interest to further explore the factors that drive the different location, phenotype, and differentiation of T_rm_ cells in various tissues and tumors.

Based on these newly generated scRNA‐seq data and bulk RNA‐seq data from TCGA researchers identified that higher ratio of T_rm_ to T_exh_ signature gene expression associated with better overall survival in NSCLC patients.^60^ Similarly, T_rm_ signature genes defined by another scRNA‐seq analysis in breast cancer samples also correlated with better prognosis in these patients.^132^ Notably, T_rm_ signature genes in breast cancer also included several inhibitory receptors that are hallmarks of T_exh_ cells.^132^ It needs to be explored whether the favorable prognosis stemmed from T_rm_ or T_exh_ cells. Nevertheless, these scRNA‐seq studies provide evidences for the important roles of T_rm_ cells in the control of solid tumor.

## 4 CONCLUDING REMARKS

Single cell transcriptomes have revolutionized the way to study the highly complex TME and provide a better understanding of various immune cell populations in this context. As illustrated in this review, scRNA‐seq based studies have unraveled the detailed characteristics of heterogeneous tumor‐infiltrating T cells in various human cancer types. By incorporating the transcriptomes and paired TCR analysis, single cell lineage tracing models, such as STARTRAC, now allow us not only to track the developmental trajectories but also the dynamic relationships of these T cells with different cellular states or tissue origins. Nevertheless, the snapshot of transcriptomic information captured by scRNA‐seq may make it less feasible for the analysis of other phenotypic parameters, such as spatial organization and epigenetic regulators. With continued advances in single cell transcriptomics and other omics technologies, we anticipate that the integrated single‐cell multi‐omics will broaden the scope of their applications in the immune system, such as providing cues that regulate the fate and cellular localization of tumor‐infiltrating T cells. With the accumulation of scRNA‐seq and other single cell omics data for baseline tumors and drug‐treated tumors, it is promising to discover tumor‐infiltrating T cell subtypes associated with treatment responses and explore their functions and relationships with other immune and stromal cells. Such information will further provide opportunities for the identification of improved biomarker strategy in the clinic to predict patient response to CPIs and the development of novel immunotherapeutic strategies to treat cancer.

The heterogeneous response of cancer patient to checkpoint blockades warrants urgent needs to address the mechanisms underlying resistance to these therapies. It remains elusive whether anti‐PD1 treatment can reinvigorate intratumoral T cells that recognize neoantigens or promote the development of new tumor Ag‐specific T cell clones. With above‐mentioned approach, one can address this question with tumor‐infiltrating immune cells collected before and on treatment. Applying scRNA‐seq analysis to characterize intratumoral immune cell changes to anti‐PD‐1 have started to emerge.^126^ Alternatively, T cells in a given cancer type but with different outcomes to checkpoint blockades can be compared at single cell level. For example, in our CRC study, we compared intratumoral T cell clusters in MSI patients versus MSS patients given these patients exhibit distinct responses to anti‐PD‐1 treatment.^151^ A new *CXCL13* ^+^ *BHLHe40* ^+^ Th1‐like CD4^+^ subset was found to be present at higher proportion in MSI tumor and may explain higher IFN‐γ level in MSI tumor and better response of these patients to anti‐PD‐1 treatment.^151^, ^152^ Other studies along similar lines are expected in the near future and these data will provide critical insight into mechanisms rendering tumors refractory to checkpoint blockade.

It is known that T cell functions can be greatly impacted by other factors and cells in TME. With further advancement of scRNA‐seq technology, combining with other single cell level techniques (such as scATAT‐seq), we foresee a great expansion of our understanding about tumor‐infiltrating immune cell heterogeneity, the intercellular interactions between intratumoral immune cell clusters, and the key regulatory pathways controlling cell fate decision in this context. These findings will shed light on the development of novel immunotherapeutic strategy to ultimately benefit cancer patients.
